# Scraps

**Published:** 1887-06

**Authors:** 


### SCRAPS

PICKED UP BY THE ASSISTANT-EDITOR.

---

**What's in a Name?**—In Boston they do not say stomach-ache, but "gastric neuralgia." But it gets there all the same.—*Life* quoted in *The Medical Record* (New York).

**Haunted.**—The Parisian journalist keeps up the tradition of satirising the professors of the art of healing. Here is his most recent effort. "Do you believe, doctor, in ghosts?"—the question is asked during a conversation on spiritualism. "My dear sir," is the reply, "how can you ask me such a question? If I believed in ghosts, I should give up my profession."

**A Medical Epitaph.**—T. C. M., in *The Cincinnati Lancet and Clinic*, says that one of the most charming epitaphs written on doctors is that over the grave of Charles de l'Ecluse (also known as Carolus Clusius), the talented medical botanist, who died in 1609:

> "Qui videt hos flores tumuli de vertice nasci,
> Hæc cineri tellus ultima dona dedit.
> O bene, quod tumulo claudatur Clusius isto!
> Qui coluit flores, floribus ille jacet."

**Scotch Candour.**—There was a bad epidemic of typhus in Glasgow when Dr. Norman Macleod was at the Barony Kirk. The wife of an old man who was down with it became very anxious about him, and sent for a minister to see him. The minister, after his interview, said to the old lady, "Haven't you been in the habit of attending a place of worship?" "Ow ay! we gang tae the Barony Kirk." "Then why did you not send for Dr. Macleod instead of for me?" "Man! dae ye think we wud sen' for sae guid a man as that? Dinna ye ken it's a bad case o' typhus?"

**A Hint for Hospitals.**—In an article in *The Hospital* on "The Registration Fee System," Lord Leigh says that at the Queen's Hospital, in Birmingham, the system is worked thus: The patients attend between 9 and 11 a.m. If the registrar is satisfied that the applicant is in a position to pay a fee of one shilling, then the fee has to be paid; but if from any cause he is unable, the fee is remitted. In 1885 the receipts from this source were £852 17s., nearly double those of 1876, when the plan was introduced. Lord Leigh recommends the general adoption of the plan.

**The Peculiar People.**—In reference to the views of these persons about medical attendance, Max O'Rell says, "I do not know there is anything very extraordinary in this belief for such a free and free-trading people as the English. A medical student, who cannot obtain his diploma by examination in England, has only to go to Scotland to obtain one without difficulty, or to America to buy one. There are plenty of people ready to trust their friends in his hands. This being so, it is not very wonderful that there should be others to be found who prefer trusting to Providence."

**At the Seaside.**—The head of a family looking for lodgings applied at a house, the appearance of which he liked. The landlady stated the number of rooms she had to let, which exactly suited him. He said he should like to see them. The landlady replied that she was very sorry she couldn't show them just then, as the other gentleman hadn't left; but if he would call in the evening he could see them. Fortunately, in the afternoon he discovered that "the other gentleman" was the dead body of the last lodger, who had died there from scarlet fever, and who was to be buried that day.

**Genius.**—In a recent discussion between a number of European psychologists, concerning the conditions which produce genius, it was maintained that it is a neurosis; and history was made to furnish numerous instances in which its appearance bore a very close relation to other distinct nervous diseases. Cæsar, Peter the Great, Napoleon, and Charles V. were epileptics; Newton's nervous system was disordered; Pascal had hallucinations; and Luther threw an ink-bottle at the Devil; Byron's parents were afflicted with ungovernable tempers; and William the Conqueror was a son of Robert the Devil. Another singular fact, brought out in discussion, is that geniuses are, as a rule, sterile. Thus, Dante, Michel Angelo, Raphael, Shakespeare, Cowper, Wordsworth, Byron, Scott, and De Quincey, either left no posterity or families which soon became extinct.—*The Medical Age.*

**Deciding on a Disease by Ballot.**—We were about three miles from Natchez, when we heard a great shouting and wailing in a negro's cabin by the roadside, and it was decided to dismount and go in. It was a tumble-down structure with but a single room, and into this were crowded sixteen or eighteen coloured people, mostly of the female sex. On a heap of straw on the clay floor was a sick woman. She was rolling her eyes and writhing as if suffering great pain. When asked what ailed her, an old white-haired negro, who was called "doctor" by the others, replied: "Dat 's jist what we doan' agree on. Some says it 's de bilyus colic, an' some says it 's de gallopin' consumpshun." "Why, man, something ought to be done for her right away!" "'Zactly, sah, 'zactly; an' Ize de doctah dat would like to take right hold ob the case." "But why don't you?" "'Kase Ize boxed up, sah. I nebber gin the same medicine fur gallopin' consumshon' dat I do for bilyus colic, an' until we kin decide on de ailment I doan' dare go ahead." We were on our horses and ready to move off when the old man came out with a happier look on his face, and said: "Gem'len, we has sorter took a vote on it, an' we has declar'd it rheumatism by one majority. Ize gwine right at it to heat the bricks an' gin her an all-night sweat."—*Pacific Medical and Surgical Journal.*

**Pathological Difficulties.**—The worst part of the hospital discipline at St. Bartholomew's about 1820 was the regulation—or rather non-regulation—of the pathological dissections. We had vast opportunities for following that branch of professional study; for many cases of organic disease were admitted in their advanced stages. Mr. Stanley, the anatomical demonstrator, was an ardent pathologist; and leave from the relatives of the deceased person was not, as in Edinburgh, a necessary condition. There was, therefore, usually a race between the relatives and the students—the former to carry off the body intact, the latter to dissect it. Thus dissection was apt to be performed with indecent—sometimes with dangerous—haste. It was no uncommon occurrence that, when the operator proceeded with his work, the body was sensibly warm, the limbs not yet rigid, the blood in the great vessels fluid and coagulable. I remember an occasion when Cullen commenced the dissection of a man who had died suddenly one hour before, and when fluid blood gushed in abundance from the first incision through the skin, made in drawing his knife from the upper to the lower end of the sternum in the usual manner. Instantly I seized his wrist in great alarm, and arrested his progress; nor was I easily persuaded to let him go on, when I saw the blood coagulate on the table exactly like living blood. At the hospital of La Charité, in Paris, a day seldom passed without at least one pathological inspection. I remember, nevertheless, having once met with a disappointment on three successive mornings. On the third I encountered at the door of the pathological rooms the following apostrophe from the "Gardien": "Encore, monsieur, point d'autopsie! Il y a depuis ces trois jours une épidémie de santé dans l'hôpital!"—*The Life of Sir Robert Christison.*

